# Which Patients Are Prescribed Escitalopram?: Predictors for Escitalopram Prescriptions and Functional Outcomes among Patients with Acute Ischemic Stroke

**DOI:** 10.3390/ijerph15061085

**Published:** 2018-05-28

**Authors:** Jarim Kim, Yerim Kim

**Affiliations:** 1 School of Communication, Kookmin University, Bugak Hall 603, 77 Jeongneung-ro, Seongbuk-gu, Seoul 02707, Korea; jrkim@kookmin.ac.kr; 2 Department of Neurology, Kangdong Sacred Heart Hospital, College of Medicine, Hallym University, Seoul 05355, Korea; 3 College of Medicine, Seoul National University, Seoul 03080, Korea

**Keywords:** antidepressant prescriptions, stroke, cerebral infarction, recovery, functional outcome, escitalopram

## Abstract

Recent studies have demonstrated that antidepressants could enhance functional recovery via neuroplasticity beyond solely treating depression. However, since Koreans typically show a greater aversion to seeking psychiatric care than citizens of Western countries, the number of antidepressant prescriptions is low. Through this study, we aim to identify the factors that lead to the prescription of antidepressants in subjects with acute ischemic stroke (AIS) in clinical practice. A total of 775 patients with ischemic stroke (IS) participated in this study from March 2010 to May 2013. We used binary logistic regression to find predictors for escitalopram prescriptions. To reveal predictors for short-term functional outcomes, we used an adjusted regression model using a propensity score. Among the 775 participants, 39 (5.03%) were prescribed escitalopram. The duration of hospital stay (odds ratio (OR) = 1.07; 95% confidence interval (CI) = 1.04–1.10) and the use of mechanical ventilation were significantly more closely related to escitalopram prescriptions as compared to non-escitalopram prescriptions (OR = 5.15; 95% CI = 1.53–17.40). The use of escitalopram, on the other hand, was not significantly associated with short-term functional outcomes (OR = 1.27; 95% CI = 0.50–3.25). Duration of hospital stay and use of mechanical ventilation were significantly related to escitalopram prescriptions.

## 1. Introduction

Stroke is a major health burden worldwide, including Korea. Because the population in Korea is aging faster than those of other Organization for Economic Cooperation and Development (OECD) countries, the stroke burden in Korea will likely increase in the near future [1]. Each year, approximately 105,000 patients experience a new or recurrent stroke, and more than 26,000 people die of stroke. The nationwide total cost for stroke care was 3,738 billion Korean won (US$3.3 billion) in 2005. Therefore, recovery from stroke is a significant issue for public health policy in Korea [1]. Globally, the point prevalence of depression is 12.9% [2]. The incidence of stroke is 850 per 100,000 for people aged 70 years and above [3]. From a public health perspective, both stroke and depression are major contributors to the burden of disease worldwide. Furthermore, both depression and stroke increase the risk of suicide [4,5].

Recovery after stroke has long been an unresolved problem for clinicians because the neurons destroyed after ischemia are not restored, despite partial neurogenesis. Recently, however, advanced neuroimaging techniques have revealed that the human brain shows spontaneous reorganization and the clinical recovery of neurological functions [6,7].

Previous studies have demonstrated that antidepressants improve functional outcomes in both depressed and non-depressed stroke patients. Both depression and stroke share similar biological mechanisms. Stroke survivors with the homozygous short variation allele genotype of the serotonin transporter-linked polymorphic region (5-HTTLPR) have higher risk of developing post-stroke depression [8]. Increased cortisol is associated with poor outcomes in stroke and depression [9,10]. Both stroke and depression are associated with an increase in inflammation [11]. Antidepressants can reduce the levels of pro-inflammatory cytokines [12]. 

These new promising approaches suggest that these drugs do more than reduce negative mood with respect to the physical consequences of stroke [6]. In contrast, non-pharmacological transitional-care interventions are not effective in reducing the mortality of stroke survivors [13]. Antidepressants might act directly on damaged neuronal circuits and modulate post-stroke reorganization.

Physicians, however, often overlook the potential positive effects of antidepressants. Compared with Western countries, antidepressant prescriptions are especially uncommon in Korea [14]. This study, therefore, aims to identify the factors that lead to antidepressant prescription among the subjects with acute ischemic stroke (AIS) in clinical practice. Escitalopram is a selective serotonin reuptake inhibitor (SSRI) with few drug interactions, and is thus suitable for stroke patients who are prescribed multiple medications [15]. To achieve this goal, this study examined the predictors for escitalopram prescriptions and effects of escitalopram on short-term functional outcomes in patients with acute ischemic stroke (AIS).

## 2. Materials and Methods

### 2.1. Study Population

A total of 823 patients with AIS or transient ischemic attack (TIA) within seven days of the onset of symptoms were consecutively enrolled in our prospective stroke registry system from March 2010 to May 2013. We excluded subjects who had a known history of depression before index events. By employing Rosner’s method [16], this study calculated the sample size based on the results of the prior study [17].

In Korea, escitalopram is one of the most commonly prescribed medicines, with relatively low side effects [18]. Because we aimed to evaluate the influence of early administration of escitalopram, we examined only those who were prescribed escitalopram for the first time during hospital admission. All subjects were instructed to receive 10 mg of escitalopram daily. The escitalopram prescriptions were based on the physicians’ judgments of either depression or neuroplasticity.

**Figure 1:**
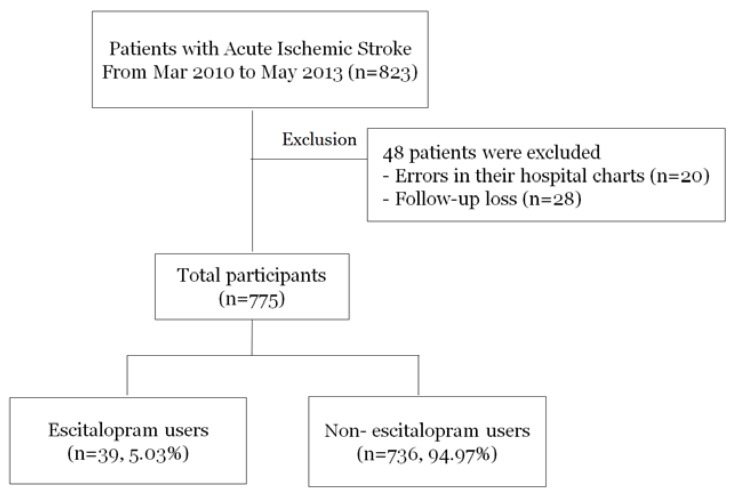
Study population.

All patients were followed through telephone interviews or medical records via outpatient care. We excluded patients with errors on their hospital charts (*n* = 20) and those lost during follow up (*n* = 28). As a result, 775 patients participated in this study, 5.03% of whom were prescribed escitalopram (Figure 1). All patients received standard, optimal medical therapy during their hospitalization. The institutional review board of Seoul National University Hospital (1009-062-332) approved the study protocol, and written informed consent was obtained from all participants or from their next of kin when the patient’s agreement was not possible.

### 2.2. Clinical Information

All patients underwent routine blood tests, neuroimaging, and cardiac studies to evaluate stroke pathophysiology. We collected baseline demographic and clinical information for all participants, including age at onset, gender, hypertension (including the prior use of antihypertensive medication), diabetes (including the prior use of an anti-diabetic medication), dyslipidemia (including the previous use of lipid-lowering agent at admission), smoking, history of stroke or TIA, and history of heart disease before the occurrence of stroke. The body mass index (BMI) was calculated as the patient’s weight (kg) divided by the square of his or her height (m). We evaluated type of meal three days after stroke onset (general diet, tube feeding, or fasting) because certain patients might be required to fast over the initial one to two days due to severe neurological deterioration or operability considerations.

We classified stroke subtypes based on the method reported in the Trial of Org 10,172 in Acute Stroke Treatment (TOAST). Initial neurological severity was assessed using the National Institutes of Health Stroke Scale (NIHSS) score at admission. Because the NIHSS scores were not normally distributed, we classified them using three levels (0–7, 8–14, or ≥15) [19]. The change in neurological severity was defined as the NIHSS score at admission minus the NIHSS score at discharge. The change in neurological severity was classified into four groups: much improved (>3), mild improved (1–3), stable (0), and deterioration (negative values). The short-term functional outcome was estimated using the modified Rankin Scale (mRS) three months after stroke onset. The three-month mRS score was obtained via an outpatient visit or structured telephone interview. We analyzed the short-term functional outcomes after dichotomizing the mRS score at three months after stroke onset, where favorable outcome was a three-month mRS score of 0–2, and an unfavorable outcome was a three-month mRS score of 3–6 [20].

### 2.3. Statistical Analyses

The distributions of the demographic data, laboratory findings, duration of hospital stay, duration of intensive care unit (ICU) stay, duration of ventilation use, and stroke subtype data based on the use of escitalopram were analyzed using χ^2^-tests or Student’s *t*-tests as appropriate. We performed a binary logistic regression analysis for escitalopram prescriptions compared with non-escitalopram users. Because this study was retrospective in its design, we used the propensity score as the conditional probability of being treated with escitalopram. We conducted an adjusted regression using the propensity score for short-term functional outcomes. The linearity assumption of the logistic regression model was examined using Box-Tidwell test and satisfied.

In this analysis, the non-escitalopram users were used as the reference group. Regarding the dependent variables, the patients with favorable outcomes (three-month mRS of 0–2) were used as the reference group in the binary logistic regression. The values for the continuous variables were expressed as the means ± standard deviations (SDs). Odds ratios (ORs) and 95% confidence intervals (CIs) are presented in the results, which were used to calculate the probability values. A probability value of ≤0.05 was considered as significant. Analyses were performed using SPSS version 21.0 (IBM, Armonk, NY, USA).

## 3. Results

### 3.1. Analysis I: Predictors for Escitalopram Prescriptions

**Figure 2:**
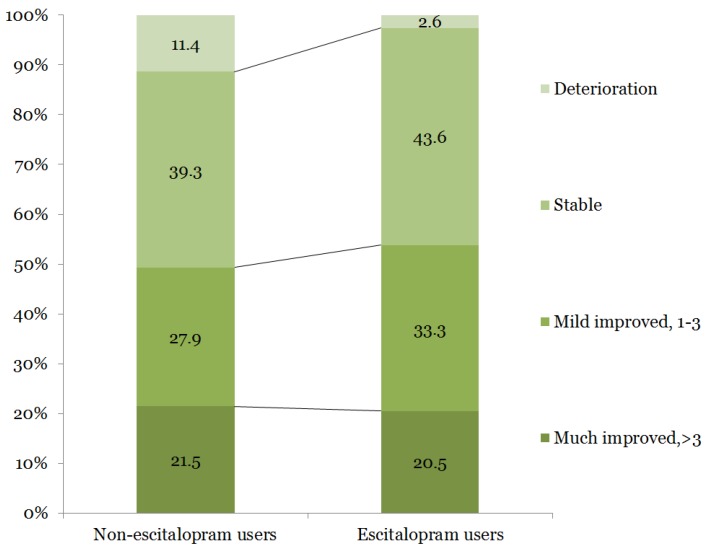
Four categories of changes in neurological severity. Change in NIHSS score = NIHSS score at admission—NIHSS score at discharge. Much improved: >3; mild improved: 1–3; stable: 0; and deterioration: negative value. NIHSS: National Institutes of Health Stroke Scale. Percentages may not add up to 100% due to rounding.

The mean age of the 775 participants was 66.8 ± 12.9 years, and 38.8% were women. The baseline demographic and clinical characteristics are shown in Table 1. Exactly 39 patients (5.03%) were classified as escitalopram users. Escitalopram users were relatively older and more frequently classified as having a cardioembolic subtype of stroke etiology than non-escitalopram users. Escitalopram users more frequently had dysphagia, tube feeding at 3 days after stroke onset, longer hospitalizations, more frequent ICU stays, longer ICU stays, more severe neurological severities, and were more to have mechanical ventilation and history of infection during the admission period. Escitalopram users were less likely to show neurological deterioration (2.6%) than non-escitalopram users (11.4%). However, this difference was not significant in terms of the change in neurological severity (Figure 2).

We conducted a binary logistic regression analysis after adjusting for multiple covariates including age, gender, duration of hospital stay, presence of mechanical ventilation, history of infection during admission, dysphagia, previous history of ischemic stroke or TIA, hypertension, diabetes, dyslipidemia, atrial fibrillation, stroke subtype, and initial neurological severity. The duration of the hospital stay (OR = 1.07; 95% CI = 1.04–1.10) and the use of mechanical ventilation were significantly more related to escitalopram prescriptions than to non-escitalopram prescriptions (OR = 5.15; 95% CI = 1.53–17.40; Table 2).

### 3.2. Analysis II: Escitalopram Prescriptions and Short-Term Functional Outcomes

Of the 775 patients, 23.2% (*n* = 180) showed poor functional outcomes (mRS score of 3–6 at three months after stroke). According to the propensity score-adjusted model, escitalopram use was not significantly associated with short-term functional outcomes (OR = 1.27; 95% CI = 0.50–3.25). Initial neurological severity was independently related to poor outcomes at 3 months after stroke onset (Table 3).

## 4. Discussion

In summary, we found that the prescription rate for an escitalopram was low (5.03%) at our hospital. We noted that hospital duration and mechanical ventilation were independent indicators for escitalopram prescriptions in our ischemic stroke patients. In addition, although these types of drugs are related to improved neurological severities during admission, the current study did not find significant associations between escitalopram prescriptions and short-term functional outcomes.

Two plausible explanations exist about the association between escitalopram and functional outcome. First, negative moods after stroke might affect the physical consequences of the rehabilitation. According to previous reports, depression is the most common psychiatric disorder, and it might contribute to post-stroke morbidity and mortality [21,22,23]. Furthermore, an important but often overlooked aspect is that depression is associated with both structural and functional changes within certain brain regions, including the hippocampus, amygdala, and prefrontal cortex [24]. The default mode network (DMN, which includes the insula, cingulate, frontal, and parietal regions) is composed of a series of interconnected cortical regions [25]. This goal-associated shifting between the DMN rest state and activation pathways appears to be disrupted in patients with depression [26]. Therefore, we hypothesize that antidepressants help patients with stroke and depression move or engage with positive external stimuli. Second, one important point to consider is that antidepressants exert an acute neuroprotective action on the ischemic brain and promote hippocampal neurogenesis [27,28]. Antidepressants, especially selective serotonin reuptake inhibitors (SSRIs), show positive effects on the recovery process among patients with ischemic stroke [7,29]. A functional magnetic resonance imaging study revealed that fluoxetine increases growth factors and other proteins such as brain-derived neurotrophic factor (BDNF). In a rat cerebral ischemic model of middle cerebral artery occlusion, SSRI-treated rats showed markedly reduced microglia activation, neutrophil infiltration, and proinflammatory marker expression. These results suggest that SSRIs imbue strong protection against delayed ischemic injury [28]. This explanation is limited with regard to our data. However, it is noteworthy that escitalopram users showed a trend of less neurological deteriorations, despite their higher initial neurological severity. 

It is noteworthy what circumstances would lead to the prescription of antidepressants in subjects with acute ischemic stroke (AIS) in real clinical practice. Escitalopram users more frequently had dysphagia, tube feeding at 3 days after stroke onset, longer hospitalizations, more frequent ICU stays, mechanical ventilation, and history of infection. All of these circumstances were correlated with poor neurological deterioration after stroke. Finding out the key predictors for escitaloptam prescriptions is important. Considering the potential positive effect of escitalopram on the damaged brain, physicians’ active interventions through escitalopram prescription might affect patients’ outcome [6,28]. In this study, we suggest that physicians should pay more attention to AIS patients with poor neurological deterioration.

It then remains to be determined why Koreans are prescribed antidepressants infrequently. A multicenter prospective cohort registry (the Adherence eValuation After Ischemic stroke Longitudinal (AVAIL) study) found that 15.4% of patients with ischemic stroke used antidepressants at 3 months [30]. The Canadian best practice recommendation found that the proportion of antidepressant use at 6 months was 32.5% [31]. However, only 27 of 423 (6.4%) patients with acute ischemic stroke in Korea were prescribed antidepressants, even though 108 were diagnosed as having either major or minor depression using the Diagnostic and Statistical Manual of Mental Disorders (fourth edition) criteria [4]. Importantly, the prescription rate for an antidepressant (escitalopram) during admission for AIS was low (5.03%) at our hospital. The dataset from the OECD countries called “Health at a Glance 2015” reported that Iceland, Australia, and Portugal were the top consumers of antidepressants, whereas Chile, South Korea, and Estonia consumed the least [1]. Koreans typically show a greater aversion to seeking psychiatric care and medical treatment than citizens of Western countries.

It should be noted that why only escitalopram was prescribed during admission for AIS in this study. In Korea, escitalopram is the most widely used drug because of low side effects [18]. According to the Clinical Research Center for Depression (CRESCEND) study in Korea, SSRIs were most commonly prescribed initially as antidepressants (48.9% for all patients). The most commonly prescribed SSRIs were escitalopram (22.4%) and paroxetine (18.7%) [18]. A recent meta-analysis demonstrated that escitalopram showed the most favorable balance between efficacy and acceptability among 12 next-generation antidepressants [32]. Because we investigated early efficacy of antidepressant prescription, only escitalopram tended to be prescribed during the relatively short admissions for AIS.

Despite the strengths of our relatively well-investigated functional outcome results, we acknowledge that the current study has several limitations. First, this is a retrospective, observational study, and thus unknown factors might have confounded our results. A null association between the use of escitalopram and neurological functional outcomes might be due to the inability to reach the minimum duration of escitalopram use to exert its effect on stroke. Future research needs to control such confounding effects in examining the relationship between escitalopram use and stroke recovery. Second, we obtained data using only prescriptions for escitalopram because it was the most commonly used and only prescribed SSRI during admission at our hospital. Therefore, we should not conclude that SSRIs have no effect on short-term outcomes. Third, patients with very mild or very severe neurological status might not be prescribed escitalopram because patients with very mild neurological severities do not need antidepressants and are discharged earlier, and physicians initially give greater focus to stabilizing the vital signs of patients with very severe neurological deterioration. These considerations might have affected our results. Fourth, the presence of obesity was not reported in this study. Obesity is a risk factor for stroke and depression [33,34] and could have confounded the results. Fifth, this study did not report the prevalence of depression in escitalopram and non-escitalopram users. A validated instrument such as the Hospital Anxiety and Depression Scale (HADS) should be used, as this scale was designed to assess for depression in medical patients [35]. Sixth, given that only approximately 5% of the enrolled patients were prescribed escitalopram, the estimation of the propensity score may not have appropriately corrected the bias issues between two groups. Finally, escitalopram was prescribed based on physicians’ judgments. Thus, the conditional probability of being treated with escitalopram differed across the sample, although we excluded patients with diagnosed depression and adjusted for the propensity score to balance the probability for escitalopram prescriptions.

## 5. Conclusions

To conclude, the effects of escitalopram on short-term functional outcomes were limited among patients with AIS. However, considering the low rate of escitalopram prescriptions and low levels of neurological deterioration, we suggest that better designed studies with larger populations be undertaken to reach more complete conclusions.

## Figures and Tables

**Table 1:** Baseline characteristics and the differences between escitalopram users and non-users.

Characteristic ^1^	Non-Escitalopram User	Escitalopram User	*p*-Value ^2^
No. (%)	736 (94.97)	39 (5.03)	
Age, years	66.6 ± 13.0	71.4 ± 10.5	0.02
Female gender, %	284 (38.6)	17 (43.6)	0.53
BMI at admission, kg/m^2^	23.74 ± 3.29	23.43 ± 3.15	0.55
Dysphagia	89 (12.1)	19 (48.7)	<0.001
Type of meals at 3 days after stroke onset			
General diet	606 (82.3)	18 (46.2)	<0.001
Tube feeding	87 (11.8)	19 (48.7)	
Fasting	43 (5.8)	2 (5.1)	
Cardiovascular risk factor			
Prior ischemic stroke	124 (16.8)	5 (12.8)	0.51
Hypertension	445 (60.5)	24 (61.5)	0.89
Diabetes	221 (30.0)	11 (28.2)	0.81
Dyslipidemia	225 (30.6)	16 (41.0)	0.17
Smoking	253 (34.4)	12 (30.8)	0.64
Atrial fibrillation	125 (17.0)	8 (20.5)	0.57
Stroke subtype			<0.05
Large artery atherosclerosis	188 (28.4)	11 (28.9)	
Small vessel occlusion	157 (23.7)	4 (10.5)	
Cardioembolic	148 (22.4)	16 (42.1)	
Undetermined	113 (17.1)	4 (10.5)	
Other determined	56 (8.5)	3 (7.9)	
Hospital stay, days	9.7 ± 7.7	21.9 ± 14.9	<0.001
ICU stay, *n* (%)	22 (3.0)	5 (12.8)	0.001
Duration of ICU stay, days	5.4 ± 6.7	12.4 ± 12.2	<0.001
Mechanical ventilation, *n* (%)	23 (3.1)	4 (10.3)	0.02
Duration of ventilator use, days	7.4 ± 9.8	4.6 ± 4.6	0.58
Infection, *n* (%)	32 (4.3)	11 (28.2)	<0.001
Initial neurological severity, median (IQR)	3 (1, 5)	4 (1, 7)	0.34
NIHSS, 0–7	601 (81.7)	32 (82.1)	0.74
NIHSS, 8–14	76 (10.3)	5 (12.8)	
NIHSS, ≥15	59 (8.0)	2 (5.1)	
Change in neurological severity			0.36
Much improved, NIHSS change > 3, *n* (%)	205 (27.9)	13 (33.3)	
Mild improved, 1 ≤ NIHSS change ≤ 3, *n* (%)	158 (21.5)	8 (20.5)	
Stable, NIHSS = 0, *n* (%)	289 (39.3)	17 (43.6)	
Deterioration, NIHSS < 0, *n* (%)	84 (11.4)	1 (2.6)	
Laboratory			
White blood cell count	7598 ± 2571	8032 ± 3236	0.31
Hemoglobin, g/dL	13.6 ± 1.9	12.8 ± 2.3	0.03
Hematocrit, g/dL	40.2 ± 5.5	38.2 ± 6.6	0.03
Fasting blood sugar, mg/dL	106.8 ± 34.7	118.0 ± 32.7	<0.05
HbA1c, %	6.4 ± 1.2	6.4 ± 1.0	0.88
Low density lipoprotein, mg/dL	102.6 ± 38.8	98.9 ± 46.0	0.62
Total cholesterol, mg/dL	172.9 ± 41.9	177.3 ± 42.8	0.53
Triglyceride, mg/dL	115.7 ± 58.3	140.9 ± 83.8	0.07
Prothrombin time	1.01 ± 0.24	0.97 ± 0.13	0.33
aPTT	32.10 ± 12.21	30.87 ± 5.75	0.62

^1^ BMI: body mass index; ICU: intensive care unit; IQR: interquartile ratio; NIHSS: National Institutes of Health Stroke Scale; aPTT: activated prothrombin time. ^2^ *p*-value indicates the difference between non-escitalopram users and escitalopram users.

**Table 2:** Binary logistic regression for prescription of escitalopram. ^1,2^

Variables	OR	95% CI	*p*-Value
Age, per 1 years	1.03	0.99–1.07	0.11
Female gender	1.51	0.63–3.60	0.36
Hospital duration, per 1 days	1.07	1.04–1.10	<0.001
ICU stay	1.75	0.50–6.11	0.38
Mechanical ventilation	5.15	1.53–17.40	<0.01
Infection	2.75	0.94–7.99	0.06
Type of meals at 3 days after stroke onset			
General diet	reference	reference	reference
Tube feeding	1.93	0.71–5.24	0.20
Fasting	1.29	0.25–6.58	0.76
Initial neurological severity			
NIHSS at admission, 0–7	reference	reference	reference
NIHSS at admission, 8–14	1.56	0.53–4.58	0.42
NIHSS at admission, ≥15	0.30	0.05–1.66	0.17
Stroke subtype			
Large artery atherosclerosis	reference	reference	reference
Small vessel occlusion	0.63	0.18–2.17	0.46
Cardioembolic	0.94	0.35–2.49	0.90
Undetermined	0.53	0.15–1.93	0.34
Other determined	0.77	0.17–3.49	0.74
Cardiovascular risk factor			
Prior ischemic stroke	0.85	0.29–2.53	0.77
Hypertension	0.79	0.34–1.80	0.57
Diabetes	0.88	0.38–2.08	0.78
Dyslipidemia	1.71	0.78–3.73	0.18
Smoking	0.98	0.40–2.45	0.97
Atrial fibrillation	1.78	0.67–4.65	0.24

^1^ Adjusted for age, gender, duration of hospital stay, mechanical ventilation, history of infection during admission, dysphagia, previous history of ischemic stroke or TIA, hypertension, diabetes, dyslipidemia, atrial fibrillation, stroke subtype, and initial neurological severity. ^2^ ICU: intensive care unit; NIHSS: National Institutes of Health Stroke Scale; OR: odds ratio; CI: confidence interval; TIA: transient ischemic attack.

**Table 3:** Binary logistic regression for unfavorable short-term functional outcome, as compared to favorable outcome. ^1,^^2^

Variables	OR	95% CI	*p*-Value
Age, per 1 years	1.00	0.98–1.01	0.79
Female gender	0.94	0.57–1.53	0.79
Initial neurological severity			
NIHSS at admission, 0–7	reference	reference	reference
NIHSS at admission, 8–14	8.55	4.93–14.81	<0.001
NIHSS at admission, ≥15	16.25	8.20–32.20	<0.001
Escitalopram use	1.27	0.50–3.25	0.61

^1^ Adjusted for age, gender, duration of hospital stay, mechanical ventilation, history of infection during admission, dysphagia, previous history of ischemic stroke or TIA, hypertension, diabetes, dyslipidemia, atrial fibrillation, stroke subtype, initial neurological severity, and propensity score for antidepressant use. ^2^ NIHSS: National Institutes of Health Stroke Scale; OR: odds ratio; CI: confidence interval.
